# Clonal B-Cell Lymphocytosis of Marginal Zone Origin: Presenting Features, Clinical Evolution and Prognostic Factors

**DOI:** 10.3390/cancers18132021

**Published:** 2026-06-23

**Authors:** Sotirios Sachanas, Gerassimos A. Pangalis, Christina Kalpadakis, Theodoros P. Vassilakopoulos, Marina P. Siakantaris, Iliana Konstantinou, Maria Moschogiannis, Xanthi Yiakoumis, Marie-Christine Kyrtsonis, Penelope Korkolopoulou, Flora N. Kontopidou, Efstathios Koulieris, Maria Psylaki, Maria K. Angelopoulou

**Affiliations:** 1 Department of Haematology, Athens Medical Center—Psychiko Branch, 11525 Athens, Greece; pangalis@med.uoa.gr (G.A.P.); mmoshogiannis@yahoo.com (M.M.); ekoulieris@gmail.com (E.K.); 2 Department of Hematology, University of Crete, University Hospital, 71003 Heraklion, Crete, Greece; xkalpadaki@yahoo.gr (C.K.);; 3 Department of Hematology, University of Athens, Laikon General Hospital, 11527 Athens, Greece; tvassilak@med.uoa.gr (T.P.V.); ilianakont@med.uoa.gr (I.K.); mkangelop@med.uoa.gr (M.K.A.); 4 1st Department of Propedeutics, University of Athens, Laikon General Hospital, 11527 Athens, Greece; mck@ath.forthnet.gr; 5 Department of Pathology, University of Athens, 11527 Athens, Greece; pkorkol@med.uoa.gr; 6 Second Department of Internal Medicine, University of Athens, “Hippocrateio” General Hospital, 11527 Athens, Greece; florakont@med.uoa.gr

**Keywords:** clonal B-cell lymphocytosis, marginal zone origin

## Abstract

**Background/Objective**: During the last two decades, several cases presenting with circulating clonal B-cells with features consistent with possible marginal zone (MZ) derivation have been described under different terminologies. The present study aims to shed more light onto the main disease characteristics and determine prognostic factors for outcome in 98 consecutive cases with clonal B-cell lymphocytosis of marginal zone origin (CBL-MZ). **Methods**: This is a multicenter retrospective analysis including 98 consecutive CD5(−) CBL cases referred to our Departments between 1999 and 2017. These cases were selected based on the presence of circulating CD5(−) clonal B-cells, irrespectively of their absolute number, without B-symptoms, organomegaly, lymphadenopathy or cytopenias or any other features consistent with a known lymphoproliferative disorder. Clinical, morphologic, biochemical, immunophenotypic, histologic and molecular features of CBL-MZ cases were analyzed. **Results**: The median absolute lymphocyte counts (ALCs) and circulating CBLs were 6.7 × 10^9^/L and 3.447 × 10^9^/L, respectively. Paraproteinemia was found in 38%. Bone marrow (ΒΜ) was involved in all but one case. MYD-88L265P mutation was positive in 11%. Two subcategories of CBL-MZ were identified: One was characterized by lower ALC/CBL, paraproteinemia, CD38 expression, BM lymphoplasmacytic morphology and more frequent MYD-88L265P mutation. The second category displayed a leukemic picture, higher frequency of CD11c expression, hypogammaglobulinemia and elevated LDH. Treatment-free survival (TFS) was 91%, and median freedom from progression (FFP) was 95.6 months. For TFS, two factors proved significant using multivariate analysis: BM infiltration ≥ 50% and elevated LDH (RR 5.6 and 5.4, respectively). Evolution to splenic marginal zone lymphoma was a rare event (5%). A novel pattern of progression emerged, namely development of cytopenias due to extensive BM infiltration without any other disease localization. **Conclusions**: CBL-MZ is an indolent lymphoproliferative disorder with excellent outcome and low probability of progression. Based on our findings, CBL-MZ represents a heterogeneous entity where the vast majority of cases remain stable or develop increasing lymphocytosis. Clinically, our data also highlight that the extent of BM infiltration and elevated LDH levels appear to be the most notable predictors for introducing therapy.

## Simple Summary

Our study retrospectively examined 98 consecutive cases of CD5(−) clonal B-cell lymphocytosis of marginal origin (CBL-MZ) referred to our Departments between 1999 and 2017. Our findings showed that the vast majority of cases either remained stable or developed increasing lymphocytosis, indicating that this entity may represent a pre-malignant state rather than a frank lymphoma. Notably, extensive bone marrow (BM) infiltration (>50%) and elevated lactate dehydrogenase (LDH) were independent prognostic factors for treatment-free survival. Furthermore, a novel pattern of progression emerged, characterized by the appearance of cytopenias due to extensive BM infiltration in the absence of other disease localization. Additionally, evolution to splenic marginal zone lymphoma was a rare event (5%). Our study sheds further light on this heterogeneous entity.

## 1. Introduction

During the last two decades, several cases presenting with circulating clonal B-cells with features consistent with possible marginal zone (MZ) derivation have been described under different terminologies [1,2,3,4,5,6,7,8]. In 2008 our group described this entity as “primary bone marrow marginal zone lymphoma”, indicating its close relationship with splenic marginal zone lymphoma (SMZL) [3,4]. Six years later in a collaborative study including 102 cases, the term clonal B-cell lymphocytosis of marginal zone origin (CBL-MZ) was proposed, which may be included in “non-CLL type monoclonal B-cell lymphocytosis” [8,9]. Based on the above study, CBL-MZ is characterized by the presence of clonal B-cells with MZ-like features in the blood and the BM in otherwise healthy individuals, without any other disease localization. As a newly emerging entity, a few aspects of CBL-MZ like require further clarification, including its classification as a separate lymphoma entity or as a pre-malignant state and its relationship to other subtypes of MZLs and lymphoplasmacytic lymphoma/Waldenstrom’s macroglobulinemia (LPL/WM). It has been suggested that CBL-MZ may represent an early phase of SMZL in the same manner as CLL-like monoclonal B-cell lymphocytosis (MBL) [9,10].

In order to shed more light on this entity, we present here data on a consecutive series of 98 CBL-MZ cases with a long follow-up observation time. We utilize the term CBL-MZ operationally, acknowledging that the biological derivation may not fully proven without tissue biology. Although CD5 expression can be found in a minority of MZL, we chose to include only CD5(−) patients in order to avoid possible atypical CLL or leukemic mantle cell lymphoma cases. The aims of this study were to: (1) analyze the clinical, morphologic, biochemical, immunophenotypic, histologic and molecular features of CBL-MZ, (2) specify the relationship of this entity to LPL/WM, SMZL or other MZL and (3) describe the clinical course, prognostic factors, disease evolution, management and outcome. We also aimed to investigate whether the proposed categories for CLL-like MBL, low and high count, are clinically meaningful in CBL-MZ [9,10,11,12].

## 2. Materials and Methods

### 2.1. Patients

This is a multicenter retrospective analysis including 98 consecutive CD5(−) CBL cases referred to our Departments between 1999 and 2017. They comprised a largely homogenous population who had visited the outpatient clinics of the participating centers. These cases were selected based on the presence of circulating CD5(−) clonal B-cells, regardless of their absolute number, without B-symptoms, organomegaly, lymphadenopathy or cytopenias or any other features consistent with a known lymphoproliferative disorder. The study was approved by the IRB Committees of our Hospitals.

### 2.2. Laboratory Investigation

All cases were evaluated with complete blood counts (CBCs); blood smear examination; biochemistry, including serum protein electrophoresis; immunofixation; virologic and certain bacteria testing.

### 2.3. Immunophenotypic/Immunohistochemical Analysis

Characterization of blood and/or BM lymphoid cells was performed by eight-color multiparameter flow cytometry. The antibody panel included: CD3, CD19, CD20, CD5, sIg, CD38, CD79b, CD23, CD10, CD200, CD11c, CD103, CD49d and κ or λ surface light chain restriction. CD20 intensity was assessed by comparison to normal B-cells according to 2006 Bethesda recommendations [13]. Not all antigens were evaluated in all patients, as indicated in the Section 3 and Table 1. Bone marrow sections were also immunohistochemically stained with antibodies specific to Immune Receptor Translocation-Associated Protein 1 (IRTA1) (FcRL4; EPR21961, rabbit monoclonal, ab239076, 1:50; Abcam plc, Cambridge, UK) and Myeloid Nuclear Differentiation Antigen (MNDA) (253A, mouse monoclonal, ab188566, 1:100; Abcam plc).

### 2.4. Computed Tomography (CT) and Endoscopy

Whole-body CT scans were performed in all cases. Upper endoscopy with multiple biopsies was available in 52 cases, along with Helicobacter pylori (Hp) testing.

### 2.5. Bone Marrow Studies

BM was available in 83 cases. BM smears and sections were assessed by expert hematologists (GAP, CK, SS, TP, MKA) and a hematopathologist (PK), respectively.

### 2.6. Cytogenetic and Molecular Studies

The presence of *MYD88 L265P* mutation was evaluated in 85 cases in the blood/BM by allele-specific PCR as described previously [14]. The sensitivity of the assay was 1.00 × 10^−3^. Fluorescence In Situ Hybridization (FISH) was performed in 40 cases using the locus-specific XL del(7)(q22q31) for del(7q) and the XCE-3 centromere DNA probe for trisomy 3, according to standard procedures [15]. In 31 cases, the IGHV mutation status was determined according to standard methodology [16].

### 2.7. Subgrouping of CBL-MZ like Cases According to the Absolute Number of Blood Clonal B-Lymphocytes

CBL-MZ like cases were subgrouped according to the number of circulating CD5(−) CBLs into low count (≤0.5 × 10^9^/L), high count (>0.5 × 10^9^/L and ≤5 × 10^9^/L) and absolute lymphocytosis (>5 × 10^9^/L).

### 2.8. Follow-Up Studies

Patients had a regular follow-up every 3–6 months, which included physical examination, CBCs and biochemical profile. Upper abdominal ultrasound was performed once a year and, in case of suspicion of increasing spleen size, the CT scan was repeated. In addition, a BM aspiration plus trephine biopsy were performed in patients who developed cytopenias during the follow-up period. Disease progression was defined by at least one of the following criteria: 1. doubling of absolute lymphocyte counts (ALCs) for cases with absolute lymphocytosis (CBL > 5 × 10^9^/L) at diagnosis, 2. increase in CBLs to >5 × 10^9^/L for cases with CBL < 5 × 10^9^/L at diagnosis, 3. doubling of paraprotein levels, 4. development of cytopenias that could not be attributed to any other cause, 5. lymph node enlargement (>1.5 cm) or development of splenomegaly [17]. Treatment was applied at the treating physician’s discretion and was not necessarily associated with biological progression. The reasons for treatment initiation were retrospectively recorded and were mainly due to the development of severe cytopenias, symptomatic disease or the appearance of autoimmune phenomena.

### 2.9. Statistical Analysis

Non-parametric tests were used for the comparison of categorical variables. Freedom from progression (FFP) was calculated from diagnosis to progression as defined above or last follow-up. Deaths from unrelated causes were censored and were not counted as events for the analysis of FFP. Patients without disease progression were censored at the time of last follow-up. Treatment-free survival (TFS) was calculated from diagnosis to treatment initiation, death or last follow-up. Overall survival (OS) and disease-specific survival (DSS) were measured from diagnosis to death from any cause or disease-related death, respectively, or last follow-up. FFP, TFS, OS and DSS along with a 2-sided 95% confidence interval (CI) were estimated according to the Kaplan–Meier method [18,19]. The following potential prognostic parameters were evaluated for FFP and TFS by univariate analysis: age, gender, WBC count, ALC, number of clonal B-cells, presence of paraproteinemia, MYD88 mutation, LDH, percentage of BM infiltration, and CD38, CD11c and CD25 expression. Survival functions were compared using the log-rank test. Significant variables at *p* values < 0.05 in the univariate analysis were entered into the multivariate analysis which was performed using the Cox proportional hazard model [20].

## 3. Results

### 3.1. Patients’ Characteristics and Referral

All 98 patients were asymptomatic at presentation with no abnormal clinical findings. Eighty-three of the 98 patients underwent bone marrow evaluation, including a bone marrow aspirate and a trephine biopsy. The remaining patients, who declined the procedure, all presented with a reversed differential white blood cell count, without symptoms or abnormal findings on physical examination. Their median age was 70 years (range, 33–90) and 54% were females. Reasons for referral included: incidental lymphocytosis (ALC > 4 × 10^9^/L) in 77%, paraproteinemia in 12%, inverted differential leukocyte count with normal ALCs in 9% and borderline anemia in 2% [attributed to β-thalassemia minor]. The median Hb level was 13 g/dL (range: 7–17). The median ALC was 6.78 × 10^9^/L (range, 1–150), while the median number of CBLs was 3.447 × 10^9^/L (range, 0.185–145). LDH was elevated in 9% of the patients. Thirty-seven percent of the 92 patients with available data presented with a normal immunoglobulin profile, 37% had paraproteinemia (59% of IgM type, 38% IgG and 3% biclonal), 24% had hypogammaglobulinemia and 2% had polyclonal hypergammaglobulinemia. Among patients with paraproteinemia, none had a BM infiltration of <10%, excluding the possibility of IgM monoclonal gammopathy of undetermined significance (MGUS). No patient was positive for hepatitis C. Among the 52 patients who underwent upper GI endoscopy, 42 had gastritis with Hp(+) in 13 of them. All HP(+) cases received anti-HP treatment with no impact on lymphocyte counts. Six patients reported a concomitant autoimmune disorder: three Hashimoto thyroiditis, one rheumatoid arthritis, one angioedema due to acquired C1 inhibitor and one primary biliary cirrhosis. Table 1 summarizes the main disease features at diagnosis.

### 3.2. Blood Morphology and Immunophenotype

The lymphomatous cells were mainly of small size (94%) admixed with medium-sized cells in 40% of the cases. Villous lymphocytes were present in 48% of the cases, while lymphoplasmacytes were rarely seen in the blood (5%).

Lymphoid cells presented intermediate (43%) to bright (52%) CD20 expression, while four cases displayed weak CD20 expression. Apart from the universal expression of CD19 and CD20, the most frequently expressed antigens were FMC-7, CD11c and CD23, which were positive in 96%, 43% and 31% of the cases, respectively. In addition, CD49d was positive in 19/19 studied cases. No case was positive for either CD5 or CD10. Monotypic expression of κ light chain was found in 65%, and surface light chain intensity was intermediate in 59%, weak in 29% and bright in 12%. Surface light chain intensity significantly correlated with CD20 intensity (*p* = 0.03). CD38 expression was highly associated with lymphoplasmacytic differentiation in the BM (*p* = 0.006), while there was a borderline significant correlation between CD11c positivity and the presence of villous lymphocytes (*p* = 0.06).

### 3.3. Bone Marrow Histologic Findings

**Figure 1:**
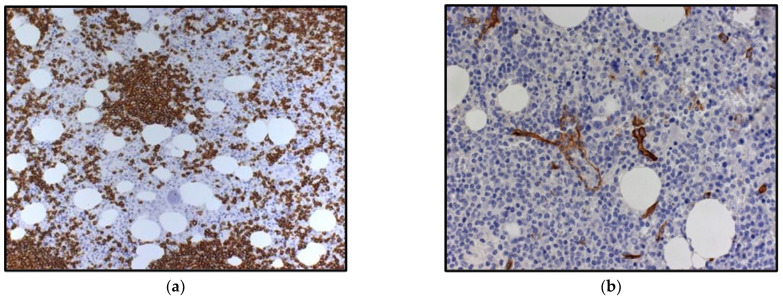
Bone marrow histology in a representative patient with CBL-MZ: (**a**) interstitial and nodular pattern of infiltration, (**b**) intrasinusoidal pattern of infiltration. Sections stained by the CD20 antibody; 250×.

In all but one case, the BM was infiltrated by small (89%)- or medium (11%)-sized lymphocytes, admixed with lymphoplasmacytes in 20%. The median percentage of BM infiltration was 30% (range, 5–90%). There was one patient without BM infiltration with an ALC of 3.9 × 10^9^/L and a CBL count of 1.326 × 10^9^/L. A single pattern of infiltration was evident in 53%, while 47% had multiple ones. Interstitial and nodular were the most common histologic patterns of involvement, while an intrasinusoidal pattern was present in one third of the cases, either alone or in combination with other patterns (Figure 1a,b). All cases were negative for CD10, CD5, annexin A, and cyclin-D1 as well as for IRTA-1 and MNDA. DBA-44 was positive in 61% of the cases.

### 3.4. Cytogenetic and Molecular Findings

MYD88 L265P mutation was detected in 9 out of 84 (11%) analyzed cases. All MYD88(+) cases were associated with paraproteinemia (*p* < 0.0001). Notably, not all MYD88-positive cases were associated with IgM paraproteinemia; in fact, two out of nine MYD-88(+) cases displayed IgG paraproteinemia.

Del(7)(q22q31) and trisomy 3 were detected in 3/41 (7%) and 5/40 (12%) cases, respectively. Interestingly, two out of three del(7q)(+) cases were also MYD-88(+) with IgM paraproteinemia, while the third patient developed splenomegaly 56 months after diagnosis.

The majority of cases [24/31 (77%)] carried mutated IGHV genes (<98% homology). In one case, 100% homology with the germline IGHV was identified. The most common IGHV genes were IGHV4-34*01 (17%), IGHV1-2*04 (13%), IGHV3-30*03 (10%) and IGHVH3-23*01 (7%).

### 3.5. Correlation Between ALC/CBL Count and Other Laboratory Parameters

Our data revealed that paraproteinemia strongly correlated with MYD88 positivity (*p* < 0.0001), a higher frequency of CD38 expression (*p* = 0.03), and prominent lymphoplasmacytic differentiation within the BM (*p* = 0.001). Interestingly, these same patients typically presented with lower baseline ALCs (*p* = 0.002) and circulating CBL counts (*p* = 0.001). Conversely, elevated LDH and CD11c expression characterized patients with higher ALC and CBL burdens (*p* = 0.02 and *p* < 0.0001, respectively). Furthermore, hypogammaglobulinemia appeared exclusively in the patient subset exhibiting lymphocytosis without an associated paraprotein component (*p* < 0.0001). Finally, the severity of BM infiltration closely reflected the absolute lymphocyte count (*p* = 0.003).

Thus, two subcategories of CBL-MZ like were revealed: one characterized by paraproteinemia, low ALCs and CBL counts, CD38 expression, lymphoplasmacytic morphology in the BM and more frequent MYD88 mutation, and the second one associated with a frankly leukemic picture, CD11c expression, hypogammaglobulinemia and a higher frequency of elevated LDH (Table 2).

### 3.6. Clinical Course, Disease Evolution, Treatment and Survival

At a median follow-up time of 42.3 months (range 1.15–223), 30 (31%) cases progressed but only 10 required treatment (Table 3). Progression included: (a) worsening of lymphocytosis in 18 (18%), with hyperlymphocytosis; (b) splenomegaly in four (4%); (c) cytopenias in four (4%); (d) lymphadenopathy consistent with the diagnosis of nodal MZL (NMZL) in one (1%); (e) increase in paraprotein levels in three (3%) cases. Median time to progression (FFP) was 95.6 months and 5-year FFP was 75%. SMZL was diagnosed during the course of the disease in five patients (5%). Apart from the aforementioned four patients who progressed with splenomegaly, one additional patient experienced rapid ALC increase, and was therefore included in this group of progression, but also developed mild splenomegaly (15 cm) and B-symptoms. Patients who progressed to SMZL had a median age of 61 years; a median WBC count, ALC and CBLs of 16.28 × 10^9^/L, 11.23 × 10^9^/L and 6.09 × 10^9^/L, respectively; and a median percentage of BM infiltration of 35% at the time of initial presentation. All of them were CD38-negative and 4/5 had villous lymphocytes. All five patients had an ALC ≥ 6 × 10^9^/L and CBL ≥ 3 × 10^9^/L and differed significantly from patients with values below these cut-offs in regard to SMZL progression (*p* = 0.043 and *p* = 0.038, respectively). Thus, these cut-offs could discriminate patients who progressed to SMZL better than the CBL value of 5 × 10^9^/L. Even so, SMZL evolution was a rare event: only 5/50 patients (10%) with an ALC ≥ 6 × 10^9^/L and CBL ≥ 3 × 10^9^/L developed SMZL versus 0% among those with values below these thresholds. Otherwise, no differences were found between cases who did or did not progress to SMZL. Only 2/5 patients who progressed to SMZL had required treatment at the time of the analysis. A total of 10 patients fulfilled criteria for treatment initiation at a median time of 6.5 years (0.5–11). These included: cytopenias in two patients, IgM paraproteinemia with hyperviscosity syndrome in one and B-symptoms with hyperlymphocytosis in one. Cytopenias were due to extensive BM infiltration in four patients, autoimmunity in three and bulky splenomegaly in one. Treatment included rituximab monotherapy in six, rituximab–alkylator chemotherapy in two and steroids in two. The details of these 10 patients are shown in Table 2. Median TFS was not reached and 5-year TFS was 90%. At the time of the present analysis, 91/98 patients were alive: 78 without any need of treatment including 3 with second neoplasia, 10 after one line of treatment, and 3 after >1 line of treatment. Seven deaths were reported with only one due to the disease, two due to secondary neoplasia and four due to other causes. Median overall survival (OS) was not reached. Five- and 10-year OS was 97% and 84%, respectively.

### 3.7. Prognostic Factors

#### 3.7.1. Treatment-Free Survival (TFS)

**Figure 2:**
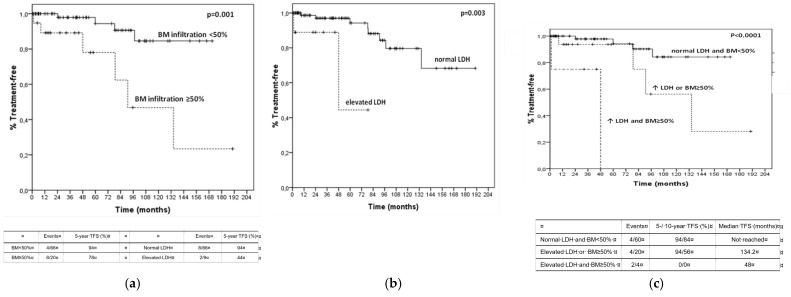
Treatment-free survival (TFS) according to (**a**) bone marrow infiltration and (**b**) LDH; (**c**) a model incorporating bone marrow infiltration and LDH.

When only the 10 patients who required treatment were considered, the following parameters proved significant for TFS in univariate analysis: BM infiltration ≥ 50% (*p* = 0.001, Figure 2a), elevated LDH (*p* = 0.003, Figure 2b), WBC count and ALC at different cut-off points. Among these, the ones remaining significant in multivariate analysis were elevated LDH and BM infiltration ≥ 50% with a relative risk of 5.6 and 5.4, respectively. Based on these two parameters, a model predicting TFS was built. Patients with both elevated LDH and BM infiltration ≥ 50% had the shortest TFS (median: 48 months), compared to those with either one of the two factors (median: 134 months), and the ones who did not have any of these two parameters (median not reached), when *p* < 0.0001 (Figure 2c).

#### 3.7.2. Freedom from Progression

**Figure 3:**
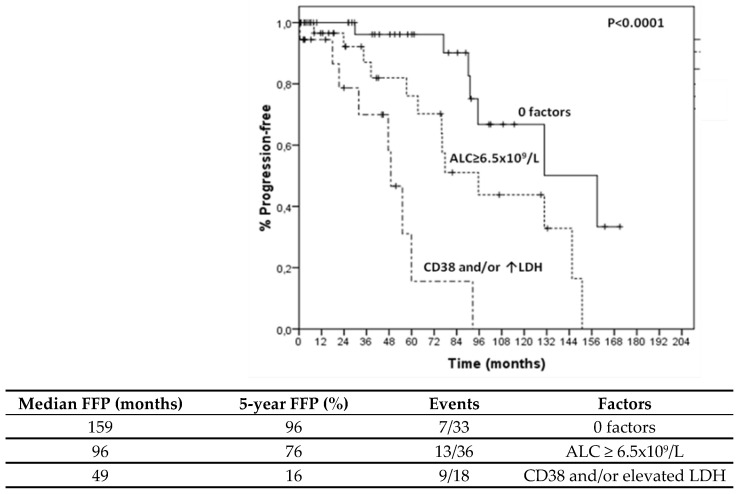
Freedom from progression (FFP) according to a model incorporating absolute lymphocyte counts, LDH and CD38 expression.

For freedom from progression (FFP), the following prognostic factors were revealed by univariate analysis: elevated LDH (*p* < 0.0001); CD38 expression (*p* = 0.006); and WBCs, ALC and CBLs at different cut-offs. Among the different cut-offs for the estimation of lymphocytosis, the most meaningful and significant value was ALC ≥ 6.0 × 10^9^/L (*p* = 0.02), which was very close to the median ALC value (6.78 × 10^9^/L). By multivariate analysis, elevated LDH, CD38 expression and ALC ≥ 6.0 × 10^9^/L remained significant with an RR risk of 12.9, 9.0 and 3.6, respectively. Thus, we constructed a model best predicting FFP based on these three factors (*p* < 0.0001). The low-risk group included patients with zero risk factors; the intermediate-risk group, the ones who had ALC ≥ 6.0 × 10^9^/L; and the high-risk group, those who had any of the remaining two factors (CD38, elevated LDH) or ≥2 factors (Figure 3).

### 3.8. Significance of Low-Count vs. High-Count vs. Absolute Lymphocytosis CBL-MZ like

In accordance with the proposed grouping of CLL-like MBL, we evaluated the clinical significance of such distinction in CBL-MZ like. The characteristics and outcome of the three groups of patients, namely *group A* (low-count < 500/μL), *group B* (high-count, 500–5000/μL) and *group C* (absolute lymphocytosis > 5000/μL), are shown in Table 4. A total of 3/98 patients belonged to group A, 62 to group B and 33 to group C.

Apart from WBCs, ALC and CBL which differed significantly by definition, the parameters that were statistically different between the three groups were CD11c expression, absolute neutrophil counts (ANCs), BM infiltration, and the frequency of evolution to SMZL. More specifically, the frequency of CD11c expression was highest in the lymphocytosis group (68%) compared to high (33%)- and low-count (0%) CBL-MZ. ANCs were significantly higher with rising ALC. The percentage of BM infiltration differed significantly between the three groups and was highest in the lymphocytosis group; however, this difference was due to the difference between the high-count CBL-MZ like and the absolute lymphocytosis. However, using the cut-off of 50% for BM infiltration, the difference between the two groups was not significant. More specifically, the three patients with low-count CBL-MZ like had BM infiltration between 30 and 40%, while among low-count CBL-MZ like patients, 22% (11/51) had BM infiltration of more than 50%, similarly to 30% (9/30) for the absolute lymphocytosis group. No difference in TFS was observed between the three groups (5-year TFS: 100%, 92% and 89% for low-count, high-count and absolute lymphocytosis groups, respectively). There was a trend of a higher frequency of SMZL evolution in patients with absolute lymphocytosis compared to low-count and high-count CBL-MZ like. Four out of five patients who evolved to SMZL belonged to the lymphocytosis group, whereas the remaining one had high-count CBL-MZ like.

## 4. Discussion

CBL-MZ like has recently been described, comprising cases with circulating clonal B-cells with features indicative of MZ origin in otherwise healthy individuals [8]. In our previous publication, we adopted the term CBL-MZ like, in order to emphasize the close relation of this entity with marginal zone lymphomas [8]. Other terms used in the literature to describe this condition include CD5(−) MBL, leukemic form of MZL or monoclonal CD5(−) B-cell expansion [1,2,5,6,7], while our group initially had proposed the term “primary bone marrow marginal zone lymphoma” [3,4]. This diversity in terminology underlines the fact that many aspects of CBL-MZ require further clarification [6,11,12]. We present here a large series of 98 consecutive patients with CBL-MZ with uniform baseline evaluation and long follow-up. We used the strict criterion of including only CD5(−) cases in order to exclude any possibility of “atypical” CLL or mantle cell lymphoma. Differential diagnosis from other B-cell lymphoproliferative disorders may be difficult, particularly from LPL/WM, splenic lymphoma with diffuse red pulp infiltration (SDRPL) and hairy cell leukemia variant (HCLv) [21,22,23,24]. In particular, CBL-MZ cases with paraproteinemia represent a diagnostic challenge in differentiating them from LPL/WM. The presence of *MYD88 L265P* mutation has been shown to be present in >90% of LPL/WM cases [14]. Nonetheless, this mutation has also been described in other low-grade B-cell lymphomas [25,26,27,28]. HCLv usually presents with hyperleukocytosis, characteristic morphology of the leukemic cells and BM histology [22]. No case in the present series carried the above features. However, it is not possible to exclude early phases of SDRPL, as its diagnosis is only established by spleen histology [21,29]. With the above restrictions, other chronic B-cell lymphoproliferative disorders were excluded based on blood smear morphology, flow cytometry, BM histology and immunohistochemistry. Moreover, the detailed work-up that was undertaken excluded the possibility of occult disease elsewhere. Thus, we introduced the term CBL-MZ like for this entity. It has to be stressed that MZ derivation is difficult to confirm without lymph node or splenic histology. Such tissues were not available by definition in our cases. However, supporting data for this terminology were the morphology, the immunophenotype of the cells and the marrow histology and immunohistochemistry. In a similar way, SMZL is also a diagnosis based on blood and bone marrow findings, without the need, any longer, for splenic tissue [30]. An alternative more “strict” term for CBL-MZ like could be CD5(−) clonal B-cell lymphocytosis not otherwise specified [CD5(−) CBL-NOS]. The most suitable terminology for this type of clonal B-cell lymphocytosis remains an open question and an evolving field. Modern molecular profiling techniques might shed light onto the true derivation of this entity [31].

A significant proportion of CBL-MZ like displays many similarities with SMZL [1,4,7,8]. Villous lymphocytes were evident in approximately half of the cases, a phenomenon that was originally described by Catovsky’s group in SMZL, who coined at that time the term “splenic lymphoma with villous lymphocytes” [32]. A study comparing Catovsky’s data with the current work would be of interest. Further indication that CBL-MZ like is closely related to SMZL is supported by the presence of an intrasinusoidal pattern of BM infiltration in 30% of our patients.

In this study significant immune disturbances were observed. More than 1/3 had paraproteinemia and 1/4 hypogammaglobulinemia, in analogy to CLL-like MBL [33].

In the present series, two categories of CBL-MZ like were identified: The first one was characterized by lower ALC/CBL, paraproteinemia, CD38 expression, BM lymphoplasmacytic morphology and more frequent MYD88 L265P mutation. The second category included cases with a leukemic picture, higher frequency of CD11c expression, hypogammaglobulinemia and elevated LDH. This latter group displayed a disease pattern closer to SMZL. However, it is not yet clear where the exact thresholds should be placed in order to correctly classify every single case within each of the two aforementioned subcategories and whether this distinction carries any clinical implications. CBL-MZ like heterogeneity is also reflected in the recently described molecular and biologic diversity among CBL-MZ like cases [8,31,34,35].

All nine *MYD88*+ cases showed paraproteinemia and they were previously reported by our group [36]. An important issue raised by these findings is whether such cases truly represent patients with underlying LPL/WM. The detection of MYD88 mutation may prove a helpful tool to discriminate these cases from the CBL-MZ like cases. However, since MYD88 L265P mutation may also be present in other low-grade B-cell lymphomas, such as SMZL, further studies are warranted to better clarify whether the presence of the mutation establishes the LPL/WM diagnosis or whether it can also be present in borderline cases associated with paraproteinemia [25,26,27]. Alternatively, BM infiltration exceeding 50% might serve as a baseline to propose a distinct lymphoma or leukemia entity, which could tentatively be termed “primary BM MZL” and would represent a specific subset within the broader CBL-MZ like spectrum.

CBL-MZ like runs an extremely indolent clinical course. One third of the cases developed signs of disease progression, but only 10% actually required treatment, at a median time of 6.5 years. The most frequent indication of treatment was cytopenias (8/10), usually associated with extensive BM infiltration, while in three cases cytopenias were of autoimmune origin. A model was constructed that could predict TFS, according to which patients with both elevated LDH and BM infiltration ≥ 50% required treatment at a median time of 4 years from diagnosis. However, the percentage of such patients was extremely low (4%).

The relationship of CBL-MZ like with MZLs and WM is not yet clear. The long follow-up of our series sheds some light onto these questions. Thus, we identified different patterns of disease evolution. Progression to SMZL was observed in only five cases (5%), which were characterized by CD38 negativity, villous lymphocyte morphology and an ALC and CBL count of ≥6 × 10^9^/L and ≥3 × 10^9^/L, respectively, at diagnosis. One case evolved to nodal MZL, while no case had concurrent gastric MALT lymphoma. A second pattern of disease evolution, observed in 18% of the cases, was progressive lymphocytosis. In this group, three patients developed hyperlymphocytosis, eventually requiring treatment. Two more infrequent patterns of disease evolution included (a) the development of cytopenias, either due to an autoimmune mechanism or extensive BM infiltration—a scenario that could be consistent with primary BM MZL—and (b) progression of paraproteinemia. These patterns of disease progression–evolution underline the heterogeneity of CBL-MZ like [31,35]. Whether extended follow-up will end up changing the incidence of these patterns remains to be seen. In the study by Xocheli et al., the percentage of SMZL evolution was reported as 15%—higher than in the present study [8]. The former report included patients from three centers, and this discrepancy might be due to different referral patterns among different institutions. On the contrary, the present analysis includes a strictly CD5− consecutive patient cohort, investigated and followed-up in a homogenous way. However, spleen involvement is difficult assess in subclinical cases, without splenomegaly. In the present analysis, spleen infiltration was excluded only by imaging studies. Therefore, cases with minimal infiltration could be missed.

CLL-like MBL is further divided into low- and high-count, with significant clinical and biological differences between the two entities [10,11,37,38,39]. No studies have been conducted so far investigating the clinical significance of such distinction in CBL-MZ like. So we aimed to investigate the differences between low-count, high-count and absolute CBL-MZ like. Of note, we identified only 3/98 patients as having low-count CBL-MZ, which is an extremely rare finding in the clinical setting [38]. Our data showed that among the three categories of CBL-MZ like, the lymphocytosis group had the highest frequency of CD11c expression (66%) and the highest incidence of progression to SMZL. In addition, we found that the threshold of ≥6 × 10^9^/L ALC and ≥3 × 10^9^/L CBL could discriminate cases developing SMZL better than the 5 × 10^9^/L CBL one. Other than that, there were no significant clinical and biological differences between the three groups. Most importantly, TFS did not differ significantly. As mentioned above, TFS can be predicted using the extent of BM infiltration and LDH, whereas CBL counts per se are insufficient for such prediction. The latter finding is in accordance with previously published data which showed that the clonal size did not affect the clinical outcome of CBL-MZ like cases [40].

Del(7q) and trisomy 3 are frequently encountered in SMZL and CBL-MZ like, although specific genomic lesions have yet to be determined [15,16,41,42,43,44].

Similarly, in the current study, FISH analyses revealed higher frequency of trisomy 3. The lower incidence of del(7q)+ cases encountered in the present cohort of CBL-MZ like patients compared to previous published data could be attributed to the fact that FISH studies were performed for the detection of (7q)22q31 chromosomal lesions alone. For this reason, further studies involving a thorough karyotypic evaluation supplemented by 3q12→qter and 7q11.2→qter FISH-targeted analyses are underway.

In accordance with published data, the pronounced frequency of hypermutated IGH genes accompanied by over-representation of the IGHV4-34 and IGVH1-2 gene rearrangements supports a similarity to SMZL [8,16,45,46]. Our study has certain limitations, including its retrospective character, incomplete data for some individuals, possible referral bias and low event count that impacts the statistical power of the multivariate analysis.

## 5. Conclusions

CBL-MZ like is an indolent lymphoproliferative disorder with excellent outcome and low probability of progression. Based on our findings, CBL-MZ like represents a heterogeneous entity where the vast majority of cases remain stable or develop increasing lymphocytosis, and may include subgroups with different biological relationships to marginal zone lymphoma and lymphoplasmacytic disorders.

## Figures and Tables

**Table 1:** Characteristics of 98 CBL-MZ patients.

Parameter		%
Females		54
Median age (range)	70 years (33–90)	
Median Hb (range)	13 g/dL (7–17)	
Median WBC (range)	12.45 × 10^9^/L (4.77–159.41)	
Median ALC (range)	6780/μL (1000–150,000)	
Median CBL (range)	3.447 × 10^9^/L (0.185–145)	
Median PLT (range)	219.5 × 10^9^/L (109–491)	
Elevated LDH	9/95	9.4
Gamma Globulins		
Normal	34/92	37
Hypogamma	22/92	24
Hypergamma	2/92	2
Paraprotein	34/92	37
— IgM	20/34	59
— IgG	13/34	38
— Biclonal	1/34	3
Median IgG levels (range)	1000 mg/dL (382–3240)	
Median IgM levels (range)	78 mg/dL (15–3859)	
Median IgA levels (range)	151 mg/dL (27–370)	
Upper endoscopy		
Normal	9/52	17
Gastritis	42/52	81
H pylori+	13/42	31
BM infiltration ≥ 30%	51/83	61
Median % of BM infiltration (range)	30 (0–90)	
MYD88 L265P+	9/84	11
CD20+	98/98	100
FMC-7+	62/65	96
CD11c+	32/75	43
CD23+	28/89	31
CD49d+	19/19	100
CD5−	98/98	100
CD10−	98/98	100
k light chain restriction	64/98	65

Hb: hemoglobin, WBC: white blood cells, ALC: absolute lymphocyte counts, CBL: clonal B-lymphocytes, PLT: platelets, LDH: lactate dehydrogenase, BM: bone marrow, H: helicobacter, CD: Cluster of Differentiation. +: positive, −: negative.

**Table 2:** Differential features of CBL-MZ subcategories.

Clinical–Biological Features	Subcategory 1	Subcategory 2
Leukemic Burden	Low ALCs and circulating CBL counts	High ALCs and circulating CBL counts
Paraproteinemia	Present	No/hypoglobulinemia frequently present
Immunophenotype	CD38 expression	CD11 expression
Genetics	Higher frequency of MYD88 mutation	Lower frequency of MYD88 mutation
Bone Marrow Morphology	Distinct lymphoplasmacytic differentiation	Standard or non-plasmacytoid infiltration
LDH	Normal or low LDH levels	Higher frequency of elevated LDH

**Table 3:** Characteristics of CBL-MZ patients who required treatment.

Sex	Age (y)	WBC/ALC/CBL (×10^9^/L)	Immunoglobulin /MYD-88 Status	LDH	ΒΜ (%)	CD38	Disease Progression, Treatment and Outcome
F	64	11.7/7.8/4.9	IgMκ/(−)	N	80	(−)	Anemia, thrombocytopenia, BM > 90% → R + Rm at 79 mo after Dx → PR → relapse → R retreatment → NR → DOD at 117 m after Dx
M	63	8.2/3.2/0.61	IgMκ/(−)	N	12	(+)	M-protein increase (50 g/L) → DRC at 60 mo after Dx → mR, Ibtutinib → NR, hyperviscosity syndrome (IgM 80 g/L) → plasmapheresis → bendamustine → NR → R-VCD → VGPR, currently alive in VGPR off treatment at 96 m after Dx
M	65	12.7/4.4/1.58	N/(−)	N	12	(−)	Lymphadenopathy → biopsy: NMZL, AIHA → R at 97 mo after Dx → CR, currently alive in CR off treatment at 111 mo after Dx
F	72	7.45/4.8/2.5	Hypergamma/(−)	N	60	(−)	Anemia, BM > 90% → R at 91 mo after Dx → CR, developed solitary bone plasmacytoma 1 year later, → RT + MPV → PD, D due to progressive MM at 132 m after Dx
F	73	16.4/10/5.4	Hypogamma/(−)	E	80	(−)	Worsening of lymphocytosis (133 × 10^9^/L), anemia, LDH increase, BM: 95% R at 48 mo after Dx → PR, R-Chl → PR, currently alive in PR off treatment at 62 mo after Dx
M	38	24.9/12.7/6.1	N/(−)	N	35	(−)	Worsening of lymphocytosis (150 × 10^9^/L), B-symptoms, BM > 95%, mild splenomegaly → R at 78 mo after Dx → PR, currently alive off treatment at 121 mo after Dx
F	69	12.7/7.5/3.8	Hypergamma/(−)	N	15	(−)	AIHA → steroids at 24 mo after Dx → CR, currently alive off treatment at 39 mo after Dx
F	77	14/6.8/1.3	IgMλ/(+)	N	50	(−)	ITP → steroids at 8 mo after Dx → CR, relapse with IgM increase (30 g/L), anemia, thrombocytopenia → DRC → NR → Ibrutinib → PR (decrease in IgM), on concurrent Etrombobag for ITP, currently alive on treatment
M	59	19.4/11.3/5	IgG-κ/NA	N	50	(−)	SMZL: massive splenomegaly, anemia, thrombocytopenia → R at 134 mo after Dx → CR, currently alive off treatment at 223 mo after Dx
F	90	159/150/145	IgG-κ + λ/NA	E	90	(−)	Anemia/thrombocytopenia 6 months after Dx → R → CR, relapsed 3 years later with AIHA, lymphocytosis (52 × 10^9^/L) → R → CR, currently alive off treatment at 41 mo after Dx

y: years; WBC: white blood cells; ALC: absolute lymphocyte count; CBL: clonal B-lymphocyte; LDH: lactate dehydrogenase; BM: bone marrow infiltration; F: female; M: male; N: normal; hypergamma: polyclonal hypergammaglobulinemia; hypogamma: hypogammaglobulinemia; NA: not available; E: elevated; R: rituximab; Rm: rituximab maintenance; Dx: diagnosis; PR: partial response; NR: no response; DOD: dead of disease; DRC: dexamethasone, rituximab, cyclophosphamide; mR: minor response; R-VCD: rituximab, bortezomib, cyclophosphamide, dexamethasone; VGPR: very good partial response; NMZL: nodal marginal zone lymphoma; AIHA: autoimmune hemolytic anemia; CR: complete response; MPV: melphalan, prednisolone, bortezomib; PD: progressive disease; MM: multiple myeloma; R-Chl: rituximab-chlorambucil; ITP: immune thrombocytopenic purpura; SMZL: splenic marginal zone lymphoma. +: positive, −: negative.

**Table 4:** Comparison of low-count vs high-count vs. absolute lymphocytosis in 98 CBL-MZ patients.

Group	A (Low-Count)	B (High-Count)	C (MZ-like Lymphocytosis)	*p*-Value
Number of patients	3	62	33	
Males	2	24	18	0.341
Median age—years (range)	71.5 (51–80)	70 (33–85)	73 (38–90)	0.507
Median WBC × 10^9^/L (range)	7.7 (6.27–8.4)	10.59 (4.77–21.83)	17.18 (10.63–159.41)	<0.0001
Median ANC × 10^9^/L (range)	3.1 (1.81–3.92)	4.2 (2–14.4)	4.9 (2.4–12.4)	0.017
Median ALC × 10^9^/L (range)	3.75 (3.7–3.82)	5.03 (1.6–11.56)	11.25 (5.148–150)	<0.0001
Median CBL × 10^9^/L (range)	0.236 (0.185–0.45)	2.315 (0.526–4.9)	6.96 (5.062–145)	<0.0001
Elevated LDH	0/3	4/57	5/33	0.384
MYD88 L265P mutation	1/2	6/51	2/28	0.17
CD23+	1/3	14/59	14/32	0.142
CD38+	1/3	7/54	1/30	0.157
CD11c+	0/3	19/58	21/31	0.002
CD103+	NA	3/23	0/13	0.174
CD25+	0/2	8/43	8/23	0.245
Paraproteinemia	2/3	26/62	6/33	0.1
Median BM infiltration in % (range)	35 (30–40)	25 (0–90)	37.5 (10–90)	0.046
Treatment indication	0	6	4	0.798
Cytopenias		5	2	
Symptomatic paraproteinemia		1	0	
Symptomatic splenomegaly		0	1	
B-symptoms		0	1	
SMZL evolution	0/3	1/62	4/33	0.09
5-year FFP (%)	100	79	61	0.127
5-year TFS (%)	100	92	89	0.643
5- and 10-year OS (%)	100/100	98/92	93/84	0.857

## Data Availability

Data available on request due to privacy.
